# The association of attentional foci and image interpretation accuracy in novices interpreting lung ultrasound images: an eye-tracking study

**DOI:** 10.1186/s13089-023-00333-6

**Published:** 2023-09-11

**Authors:** Matthew Lee, Janeve Desy, Ana Claudia Tonelli, Michael H. Walsh, Irene W. Y. Ma

**Affiliations:** 1 Division of General Internal Medicine, Department of Medicine, University of Calgary, 3330 Hospital Drive NW, Calgary, AB T2N 4N1 Canada; 2 UNISINOS University, Hospital de Clinicas de Porto Alegre, Porto Alegre, Brazil; 3 W21C, University of Calgary, Calgary, AB Canada

## Abstract

It is unclear, where learners focus their attention when interpreting point-of-care ultrasound (POCUS) images. This study seeks to determine the relationship between attentional foci metrics with lung ultrasound (LUS) interpretation accuracy in novice medical learners. A convenience sample of 14 medical residents with minimal LUS training viewed 8 LUS cineloops, with their eye-tracking patterns recorded. Areas of interest (AOI) for each cineloop were mapped independently by two experts, and externally validated by a third expert. Primary outcome of interest was image interpretation accuracy, presented as a percentage. Eye tracking captured 10 of 14 participants (71%) who completed the study. Participants spent a mean total of 8 min 44 s ± standard deviation (SD) 3 min 8 s on the cineloops, with 1 min 14 s ± SD 34 s spent fixated in the AOI. Mean accuracy score was 54.0% ± SD 16.8%. In regression analyses, fixation duration within AOI was positively associated with accuracy [beta-coefficients 28.9 standardized error (SE) 6.42, *P* = 0.002). Total time spent viewing the videos was also significantly associated with accuracy (beta-coefficient 5.08, SE 0.59, *P* < 0.0001). For each additional minute spent fixating within the AOI, accuracy scores increased by 28.9%. For each additional minute spent viewing the video, accuracy scores increased only by 5.1%. Interpretation accuracy is strongly associated with time spent fixating within the AOI. Image interpretation training should consider targeting AOIs.

**Supplementary Information:**

The online version contains supplementary material available at 10.1186/s13089-023-00333-6.

## Background

Point-of-care ultrasound (POCUS) can be used at the bedside when assessing patients with heart failure/acute dyspnea to increase diagnostic accuracy [1, 2] and provide important prognostic information [3–6]. The need to incorporate POCUS into the practice of internal medicine is increasingly recognized internationally [7, 8]. However, POCUS skills are complex to teach, involving image acquisition, interpretation, and clinical integration [7–9]. Despite image interpretation being a fundamental skill, few studies exist to guide educators on how to teach it [10]. In diagnostic imaging studies, eye-tracking technology has provided educators with a better understanding of what the image interpretation task involves and its associated errors [11–13]. The majority of these studies were on radiologists interpreting radiographs and computed tomography. Few were on ultrasound. Where eye-tracking studies were conducted on POCUS [14–17], differences in eye movement between experts and novices were noted. However, the relationship between interpretation accuracy and eye movement remains undefined. We hypothesize that POCUS interpretation accuracy is related to the learners’ attentional foci on the areas of interest (AOI) relevant to the diagnosis. If this relationship proves true, educators could consider targeting training to AOIs in their educational interventions for those learning image interpretation.

## Methods

Between January 2020 and January 2021, we invited a convenience sample of 14 internal medicine residents with any prior lung ultrasound (LUS) training to participate in this cross-sectional study. We excluded those with no prior LUS training as tracking uninformed eye movements during image interpretation may not yield helpful information.

After performing eye-tracking calibration in a seated position, consenting participants viewed and interpreted 8 LUS videos on a standardized laptop (Asus ROG Strix, GL503V) with an eye-tracking system (Tobii Tech, Danderyd, Sweden) mounted on the laptop. Eight videos were created from 6 s cineloops from our program’s anonymized teaching bank. These cineloops were played in a continuous loop for 30 s, portraying the following common LUS findings: normal lung (× 2), absent lung sliding, pleural effusion, mirror image artifact with a negative spine sign, pleural irregularity with B-lines, M-mode demonstrating absent lung sliding, and presence of B-lines and A-lines. Each video is accompanied by 1–3 questions regarding the findings and diagnosis (See Additional file 1). Participants were instructed to read the paper-based questions for each video prior to viewing the video, so that they are aware of what findings to anticipate. Participants had the option to exit the video early or view the video one additional time, within 1 min (max allotted duration 2 min).

### Defining areas of interest (AOI)

AOIs for each ultrasound video were defined as areas on the ultrasound image that required evaluation to rule in or rule out a specific finding. For example, evaluation of the spine in the far field of a coronal image of the lung base is important to rule in or rule out a pleural effusion and examination of the pleural line is important to determine if pleural sliding is present [18]. AOIs for each video were mapped independently in March 2021 by two experts (IM, JD), both certified by the American Registry for Diagnostic Medical Sonography. Three discrepancies in AOI mapping were resolved by discussion. One discrepancy involved evaluation of the lung zone labelling for a normal lung cineloop and a second discrepancy involved evaluating the depth scale in a cineloop for B lines. Post discussion, experts agreed that neither were definitively critical to the cineloops’ diagnosis. The third discrepancy involved evaluation of the far field findings deep to a non-sliding pleura, which was agreed upon to be an important area to evaluate. Both experts were blinded to the participant data, which was collected by the resident investigator (ML). The AOI were then externally validated using eye movement data of a third expert (ACT) external to our institution, whose eye movements were captured in September 2019 during a site visit.

### Outcome variables

**Fig. 1:**
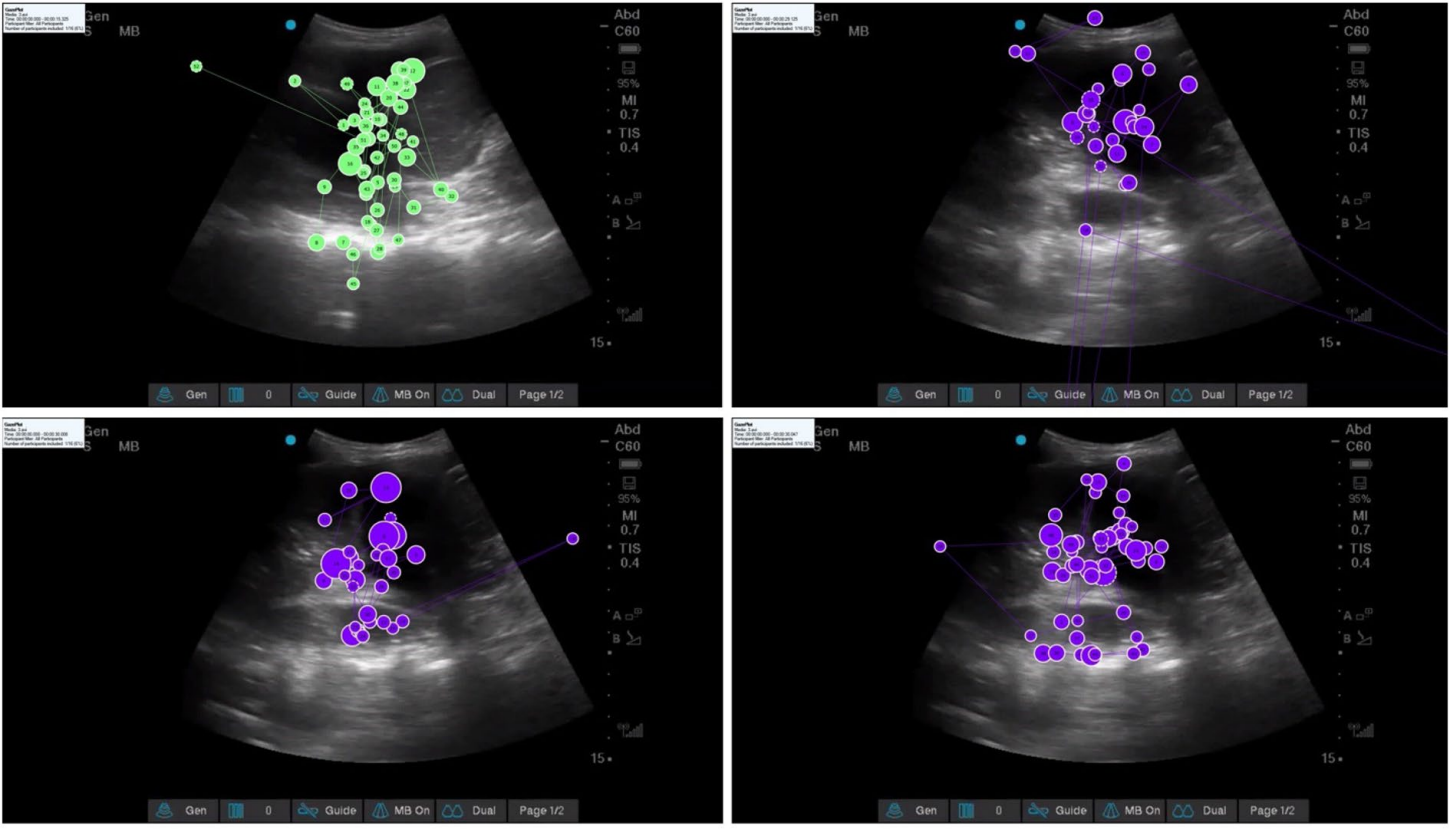
Gaze plots of expert (green, top left) and novices (purple) in identifying the presence of a positive spine sign and the pleural effusion. Top right: gaze plot of a novice who scored 0% on the video with minimal gaze on the spine. Bottom left: gaze plot of a different novice who scored 50% on the video. Bottom right: gaze plot of a third novice who correctly identified both findings and scored 100%

Total fixation duration was defined as the duration of all fixations within the AOI, (I-VT filter, default settings, minimum fixation duration of 60 ms, User’s manual Tobii Studio, version 3.4.8, 2017, pp. 54–57). Total time spent viewing the videos was time spent both within and outside of AOI. Gaze plots were created using Tobii Studio software and examined qualitatively (Fig. 1).

Accuracy score was calculated as the number of correct responses on image interpretation (out of 15), presented as a percentage. Validity evidence for the questionnaire was evaluated in two ways. First, in September 2019, for content validity, the questionnaire was reviewed and completed independently by two education experts (JD, ACT) not involved in test construction; both scored 100%. Second, we evaluated the internal reliability of the questionnaire using Cronbach’s alpha (alpha = 0.68).

### Statistical analyses

Standard descriptive statistics are reported. The independent association between eye-tracking variables and accuracy score was explored using univariate linear regression analyses. A two-sided *p* value < 0.05 was considered to indicate statistical significance. All analyses were performed using SAS version 9.4 (SAS Institute Inc., Cary, NC) and STATA 17.0 (StataCorp, College Station, TX).

## Results

All invited internal medicine residents completed the study (n = 14). However, eye tracking for four participants (29%) was not captured by the system and the data for these were excluded. Of the remaining 10 participants, five (50%) were female; all ten were first year residents. Nine (90%) reported using POCUS for under 1 year, while one (10%) reported 1–2 years of POCUS use.

Participants spent an average of 8 min 44 s [standard deviation (SD) 3 min 8 s] viewing the videos, of which, the average total fixation duration in AOI was 1 min 14 s ± SD 30 s. Mean accuracy score was 54.0% ± SD 16.8% (range 33.3–80.0%).

**Fig. 2:**
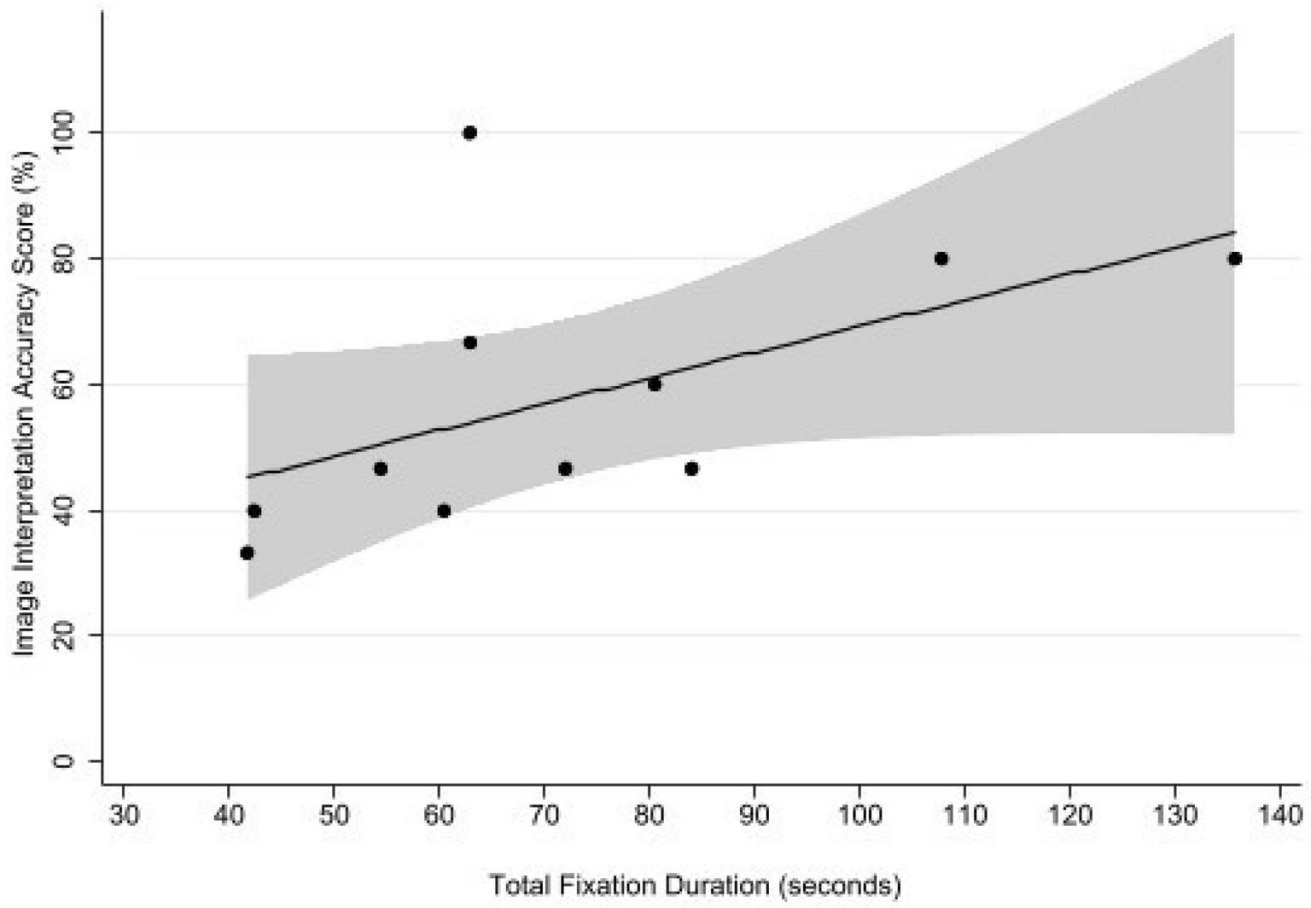
Scatter plot of image interpretation accuracy score, presented as a precent, vs. total fixation duration within areas of interest (seconds), with fitted line shown in black, and confidence interval for the mean shown in gray

Total fixation duration was significantly associated with accuracy score [Beta-coefficients (*β*) 28.9 standardized error (SE) 6.42, *P* = 0.002 for fixation duration). Total time spent viewing the videos was also associated with accuracy (*β* = 5.08, SE 0.59, *P* < 0.0001), but less so than total fixation duration. Figure 2 illustrates representative gaze plots, demonstrating qualitative differences between gaze plots of an expert and participants.

## Discussion

In this eye-tracking study on medical learners, total time spent fixating in the AOI as well as time spent viewing the videos were associated with interpretation accuracy. For every additional minute spent fixating in the AOI, accuracy score increased by 28.9%, while for every additional minute spent viewing the videos in general resulted in a score increase of only 5.1%. Our results support the hypothesis that accuracy is associated with attentional foci within the AOI.

Our results extend prior studies on eye tracking in POCUS, where most studies explored expert-novice differences. In studies on ultrasound-guided regional anesthesia, one found that novices spent more gaze time outside the AOI than experts [16]. In another study, fixation patterns differed qualitatively between one expert and one novice [17]. Two studies evaluated the interpretation of abdominal free fluid and both identified significant differences between experts and novices in their fixations in AOI [14, 15]. While helpful, identifying expert-novice differences may not be sufficient validity evidence [19]. Our study adds to this body of literature by demonstrating an additional measure of validity evidence: relations to other variables [20, 21], namely, interpretation accuracy.

How can eye-tracking data assist an educator? From an assessment perspective [20, 21], eye-tracking data can provide evidence for response process of the trainees. From a training perspective, eye-tracking data may provide feedback to learners [22], by demonstrating where errors in attention may lie. Prior studies on non-ultrasound imaging suggest that training using eye movement feedback data may increase interpretation accuracy [23, 24] and improve decision time [23, 25]. One randomized study training learners where to look in ultrasound videos using eye movement technology found higher interpretation accuracy [26]. For programs without eye-tracking technology, potentially learners may still benefit from being taught key AOIs for learners to pay attention to during image interpretation.

Our study has some limitations. This is a single institution study, which limits the generalizability of our conclusions. Second, despite finding a significant association, our study has a small sample size, including the loss of four participants’ data as their videos and eye movement data failed to capture despite completing the study. Third, our questionnaire’s internal reliability was 0.68, lower than the frequently cited threshold of 0.7 [27]. It is possible that interpretation competence is multidimensional [28] which would account for the low internal reliability. Alternatively, a longer questionnaire may be needed to demonstrate a higher internal reliability. Fourth, one of the participants had more POCUS experience than the remaining cohort. Reassuringly, however, by removing this participant’s data, our study conclusions did not materially change. Total fixation duration remained significantly associated with accuracy score (*β* = 25.5, SE 6.79, *P* = 0.007). Total time spent viewing the videos also remained significantly associated with accuracy (*β* = 4.68, SE 0.60, *P *= 0.0001).

## Conclusions

For novices interpreting LUS videos, total time spent fixating in the AOI was strongly and positively associated with interpretation accuracy. Novices may benefit from explicit instructions on key areas to look during image interpretation.

### Supplementary Information

**Additional file 1: ** Supplement 1, Eye Tracking Questions.

## Data Availability

The data sets used and/or analyzed during the current study are available from the corresponding author on reasonable requests.

## Abbreviations

AOI: Area of interest
LUS: Lung ultrasound
POCUS: Point-of-care ultrasound
SD: Standard deviation
SE: Standardized error
