# Supplementary material for: The association of attentional foci and image interpretation accuracy in novices interpreting lung ultrasound images: an eye-tracking study
Source: Ultrasound J. 2023 Sep 11;15:36. doi: 10.1186/s13089-023-00333-6 (PMC10495286; doi:10.1186/s13089-023-00333-6)
Supplement: Supplementary file 1 — Additional file 1: Supplement 1, Eye Tracking Questions. [file 13089_2023_333_MOESM1_ESM.doc]

Additional file 1: Supplement 1, Eye Tracking Questions

Eye Tracking Questions ID:__________________

**Please read over the following 3 questions. You will then be asked to watch a 30-second video, which you may exit anytime from the video if you feel you’re done. After you answer the 3 questions, if you need to, you may watch the 30-second video one more time. At any point you feel you have all the information you need, you can exit the video (you do not need to watch all 30 seconds), but you will not be allowed to watch the video more than twice.**

1. Which findings are shown on this video on the anterior right chest (please check all that apply):

A lines

B lines

Lung sliding

Consolidation

Pleural effusion

2. If you had to make one change to improve this video, what maneuver might you try? (choose one)

Increase depth

Increase gain

Change to a phased array transducer

Fan/tilt the transducer

None of the above (no maneuvers needed)

3. How would you rate the quality/acceptability of this video for evaluating for the presence/absence of B lines?

Completely UNACCEPTABLE

Somewhat UNACCEPTABLE

Mostly ACCEPTABLE (i.e. meets minimal criteria)

Completely ACCEPTABLE

Completely ACCEPTABLE and OPTIMAL

**Video 2. Please read over the following 3 questions. The same instructions applies, you will have 2 maximum opportunities to watch the video. You may exit the video anytime.**

1. Which findings are shown on this video (please check all that apply):

Irregular pleura

Absence of lung sliding

B lines

Pleural effusion

Air bronchograms

2. Does this patient have a pneumothorax at this site? (Choose best option)

Yes, definitely

Yes, possibly

No, definitely NOT

None of the above. Technically limited scan, does not meet minimal criteria

3. How would you rate the quality/acceptability of this video for evaluating for the presence/absence of lung sliding?

Completely UNACCEPTABLE

Somewhat UNACCEPTABLE

Mostly ACCEPTABLE (i.e. meets minimal criteria)

Completely ACCEPTABLE

Completely ACCEPTABLE and OPTIMAL

**Video 3. Please read over the following 3 questions. The same instructions applies, you will have 2 maximum opportunities to watch the video. You may exit the video anytime.**

1. Which findings are shown on this right upper quadrant clip (please check all that apply):

Spine sign

Lung point

Shred sign

Liver lung point

2. What pathology is seen on this video?

Pericardial effusion

Ascites

Pleural effusion

Pneumothorax

3. How would you rate the quality/acceptability of this video for evaluating for the presence/absence of pleural effusion?

Completely UNACCEPTABLE

Somewhat UNACCEPTABLE

Mostly ACCEPTABLE (i.e. meets minimal criteria)

Completely ACCEPTABLE

Completely ACCEPTABLE and OPTIMAL

**Video 4. Please read over the following 2 questions. The same instructions applies, you will have 2 maximum opportunities to watch the video. You may exit the video anytime.**

1. What finding(s) is/are seen on this video (please choose all that apply)?

A lines

B lines

C lines

Z lines

2. How would you rate the quality/acceptability of this video for evaluating for the presence/absence of B lines?

Completely UNACCEPTABLE

Somewhat UNACCEPTABLE

Mostly ACCEPTABLE (i.e. meets minimal criteria)

Completely ACCEPTABLE

Completely ACCEPTABLE and OPTIMAL

**Video 5. Please read over the following 3 questions. The same instructions applies, you will have 2 maximum opportunities to watch the video. You may exit the video anytime.**

1. What finding(s) is/are seen above the diaphragm (please choose all that apply)?

Consolidation

Pleural effusion

Artifact

Atelectasis

2. Which upper abdominal quadrant is visible in this video?

Right

Left

Cannot determine based on clip – could be either right or left

**Video 6. Please read over the following 2 questions. The same instructions applies, you will have 2 maximum opportunities to watch the video. You may exit the video anytime.**

1. What findings are present on this clip (choose all that apply)?

Consolidation

Lung point

Irregular pleura

A lines

B lines

2. How would you rate the quality/acceptability of this video for evaluating for the presence/absence of B lines?

Completely UNACCEPTABLE

Somewhat UNACCEPTABLE

Mostly ACCEPTABLE (i.e. meets minimal criteria)

Completely ACCEPTABLE

Completely ACCEPTABLE and OPTIMAL

**Video 7. Please read over the following 1 question. The same instructions applies, you will have 2 maximum opportunities to watch the video. You may exit the video anytime.**

1. Is lung sliding present based on this M-mode video?

**Yes, definitely,** pleural sliding is present

Yes, pleural sliding is **probably** present

No, pleural sliding is **definitely absent**

Cannot determine pleural sliding based on this video

**Video 8. Please read over the following 2 questions. The same instructions applies, you will have 2 maximum opportunities to watch the video. You may exit the video anytime.**

1. What findings are present on this video (choose all that apply)?

Consolidation

Lung point

A lines

B lines

Z lines

1. How would you rate the quality/acceptability of this video for evaluating for the presence/absence of B lines?

Completely UNACCEPTABLE

Somewhat UNACCEPTABLE

Mostly ACCEPTABLE (i.e. meets minimal criteria)

Completely ACCEPTABLE

Completely ACCEPTABLE and OPTIMAL
